# Data on how job satisfaction and perceived value of CSR demotivate undesirable job habits during a crisis

**DOI:** 10.1016/j.dib.2022.108520

**Published:** 2022-08-05

**Authors:** Ut Lon Im, Wilson Cheong Hin Hong, Erdan Ma, Cindia Ching Chi Lam, Leyi Zhao

**Affiliations:** a School of Tourism Management and School of Hospitality Management, Macao Institute for Tourism Studies, Colina de Mong Ha, Macao S.A.R., China; b School of Tourism and Hospitality Management, Yunnan University of Finance and Economics, 237 Longquan Road, Kunming, Yunnan 650221, China; c School of Continuing Education, Macao Institute for Tourism Studies, Colina de Mong Ha, Macao S.A.R., China

**Keywords:** Crisis, Corporate Social Responsibility, Stress, Habit Change, Escape Habit, Pandemic

## Abstract

This dataset was collected one year after the outbreak of the COVID-19 pandemic from December 2020 to January 2021 in Macao Special Administrative Region, China. The aim was to investigate the behaviour changes of employees working in the service industry under the stress of pandemic, and the roles of job satisfaction and corporate social responsibility (CSR) as individual and organizational mediators respectively at alleviating undesirable job habits. Data collection was done both offline and online due to stringent pandemic preventive measures during the collection period. Respondents of this survey included employees in different sectors of the service industry, for instance, travel agencies, hotels, casinos, food and beverage, and retails. A total of 895 responses were collected, in which 23 responses were removed during data cleaning, and 872 responses were retained in the dataset. The dataset can serve as the base of reference for future studies on employee habit change, both within and under crisis. It can also serve as a reference for future studies that examined effective ways of minimizing negative job habit changes that ensued from crises.

## Specifications Table

**Table:** 

Subject	Tourism, Leisure and Hospitality Management
Specific subject area	Management of job habits of employees in tourism and hospitality industries under crisis
Type of data	Table
How the data were acquired	Digital and Printed Survey
Data format	Raw Filtered
Description of data collection	Data were collected both online and offline due to pandemic preventive measures. An offline survey was conducted by trained surveyors at spots with major traffic. The data collection was done at different times of the day and different days of the week. The online questionnaire was developed using Qualtrics and disseminated through service industry associations and tourism and hospitality university alumni groups. Respondents who could not pass the screening questions were excluded.
Data source location	Respondents of this study were employees in tourism and hospitality industries, including travel agencies and related, hotels, casinos, food and beverage, retail, as well as other tourism services. City/Town: Macao SAR Country: China
Data accessibility	Repository name: Mendeley Data Direct URL to data and questionnaire: https://data.mendeley.com/datasets/5s9vcx26h3/4
Related research article	C.C.C. Lam, E. Ma, U.L. Im, W.C.H. Hong, L. Zhao, A new dimension in the value of corporate social responsibility: demotivating undesirable job habits during crisis, Journal of Hospitality and Tourism Management (In press). [1]

## Value of the Data

• The data provided are samples of employees in various sectors of the tourism and hospitality industry in Macao, where the yearly tourist arrival was once over 39 million [2] and the whole tourism industry contributed to 80% of the public income [3] before the pandemic. Therefore, the data has a significant representation of the situation of tourism and hospitality employees in a well-developed destination under the stress of a crisis.
• The dataset will benefit those studying the management of employee behaviour and crisis management, in the context of tourism and hospitality or service industry.
• The dataset can serve as the base of comparison in future studies of employee habit change under crises.

## 1 Data Description

The survey consists of six major parts. Demographics aside, the first part inquired the level of influence of the pandemic on work. A 5-point Likert scale was applied to measure the level of influence of the pandemic on respondents’ jobs (1 – Not affected to 5 – significantly affected) while a dichotomous scale was applied to check whether respondents encountered a specific change at work (1 – No and 2 – Yes). The second part was about demographic information. The rest were adapted or consolidated from past literature. These include Depression Anxiety Stress Scale (DASS-21) [4] where a 4-point Likert scale was applied to measure the applicability of the descriptions in the DASS-21 scale (1 – not applicable to 4 – most applicable); change of work habit [5], [6], [7], [8], job satisfaction [9] and perceived value of Corporate Social Responsbility [CSR, 10] where a 7-point Likert scale was applied to measure the agreement of statements (1 – strongly disagree to 7 – strongly agree).

There are a total of 872 samples recorded by rows. All survey items were labelled in the first row of the data spreadsheet, and all data were coded in numbers. Table 1 presents the survey items and the corresponding labels used. Table 2 presents the code used in recording responses in demographic variables. Table 3 presents a summary of the demographic of the respondents.

**Table 1:** Details of survey items and the corresponding coding.

Label	Survey Item	Responses
**Demographic**		

Industry	Which industry (within tourism) do you work for?	Options
Gender	Gender	Options
Age	Age Group	Options
Education	Education	Options
Martial	Marital Status	Options
Income	Monthly Income	Options

**Effect of pandemic at work**		

PanImpactLv	How is your job affected by COVID-19?	5-point Likert
PanImpact1	Do you encounter pay cut during pandemic?	Dichotomous
PanImpact2	Do you encounter benefit cut during pandemic?	Dichotomous
PanImpact3	Do you encounter compulsory no-pay leave during pandemic?	Dichotomous
PanImpact4	Do you encounter suspension during pandemic?	Dichotomous
PanImpact5	Do you encounter duty assigned beyond your post during pandemic?	Dichotomous
PanImpact6	Do you encounter relocation to other posts during pandemic?	Dichotomous
PanImpact7	Do you encounter worsened relationship with colleagues during pandemic?	Dichotomous

**DASS-21**		

S1	I found it hard to wind down	4-point Likert
S6	I tended to over-react to situations	4-point Likert
S8	I felt that I was using a lot of nervous energy	4-point Likert
S11	I found myself getting agitated	4-point Likert
S12	I found it difficult to relax	4-point Likert
S14	I was intolerant of anything that kept me from getting on with what I was doing	4-point Likert
S18	I felt that I was rather touchy	4-point Likert

**Work habit change (self-discipline)**		

SD1	I have never thought of absence at the peak of the pandemic	7-point Likert
SD2	I have never intended to leaving the job at the peak of the pandemic	7-point Likert
SD3	have never asked others to cover for me	7-point Likert
SD4	I have never made excuse to shirk work	7-point Likert

**Work habit change (escape)**		

E7	I have higher frequency of lateness than before	7-point Likert
E8	I have higher frequency of absence than before	7-point Likert
E10	I make excuse to leave the post	7-point Likert

**Work habit change (anti-crisis behaviour)**		

AC20	I increase the frequency of stopping customers gathering	7-point Likert
AC21	I increase the frequency of stopping the behavior which is against pandemic preventive measures	7-point Likert
AC22	I care more about the health of my colleagues	7-point Likert

**Job satisfaction**		

JS1	I find real enjoyment in my job	7-point Likert
JS2	I am fairly well satisfied with my job	7-point Likert
JS3	I am enthusiastic about my work	7-point Likert
JS4	Overall, I am satisfied with my work	7-point Likert
**Perceived value of CSR**		

CSR1	I think the company should disclose corporate social responsibility (CSR) information	7-point Likert
CSR2	I think that the disclosure of CSR information is valuable to improve company reputation	7-point Likert
CSR3	I think that the disclosure of CSR information is valuable to increase employee loyalty	7-point Likert
CSR4	I think that the disclosure of CSR information is valuable to improve employee ethics	7-point Likert

**Table 2:** Demographic variables.

***Industry***			
1. Travel Agency related	2. Hotels	3. Casino / Junket	
4. Food and Beverage	5. Retail	6. Other tourism services	
***Gender***			
1. Male	2. Female		
***Age group***			
1. 16-24	2. 25-34	3. 35-44	
4. 45-54	5. 55-64	6. 65 or above	
***Education***			
1. Primary school or below	2. Secondary school	3. Diploma	
4. Bachelor	5. Postgraduate		
***Marital Status***			
1. Single	2. Married	3. Separated/divorced	4. Widowed
***Monthly income (MOP)***			
1. $3,999 or less	2. $4,000 - $7,999	3. $8,000 - $11,999	4. $12,000 - $15,999
5. $16,000 - $19,999	6. $20,000-$29,999	7. $30,000-$39,999	8. $40,000 or more

**Table 3:** Demographics of Participants (%).

Male	61.47	From Macao S.A.R.	66.4
Female	38.53	Mainland China	28.56
		Hong Kong S.A.R.	2.52
16-24	12.27	Other regions	2.52
25-34	40.14		
35-44	25.23	Primary education or lower	3.91
45-54	17.66	Secondary education	36.13
55 or above	4.7	Diploma / College	19.45
		Bachelor's degree	38.67
Single	43.05	Master's degree or higher	1.84
Married	53.39		
Others	3.56	Management	43.22
		Non-management	56.78

## 2 Experimental Design, Materials and Methods

Purposive sampling was applied in data collection in order to ensure the employees in different key sectors of the tourism and hospitality industries are included. The survey contents were prepared in English and translated to Chinese with multiple reviews by experienced translators. A pilot study was carried out to assure the clarity of wordings. Data were collected both online and offline from December 2020 to January 2021 in Macao SAR, China.

The offline survey was conducted by trained surveyors at 12 designated locations, including the central business district, major bus hubs, intersections of main roads, markets, leisure areas, and employee shuttle bus stops of integrated resorts. These spots cover different districts of the city. The data collection was done at different times of the day and different days of the week. Spot checks were also carried out to ensure the quality of data collection.

Considering the stringent preventive measures against the pandemic, data collection was also conducted online. Similar to offline face-to-face data collection, screening questions were in place to ensure the respondents meet the requirement of this survey.

The collected data were reviewed and cleaned for three rounds to identify potential input errors or data issues before running any analysis in statistical software. Stringent filtering measures were imposed to ensure the validity of the data, like surveys taking less than 120 seconds to complete or having equal or more than 5% of missing information were excluded from the analysis. 23 samples out of the total 895 collected samples, as a result, were excluded from further analysis. Before running any analysis on the model, the data were first checked for reliability, normality and multicollinearity. Scale items were factor analysed and those with low factor loadings were excluded. The measurement model was then tested by structural equation modelling (SEM) with confirmatory factor analysis (CFA). Composite reliability, construct validity, convergent validity and discriminant validity were examined. The best fit final model was then identified.

## Ethics Statements

All ethical considerations have been addressed. The research design only involves survey. Therefore, prior ethics approval is not required. Informed consent of all respondents have been obtatined. Any reference to individual respondents has been deleted.

## CRediT authorship contribution statement

**Ut Lon Im:** Funding acquisition, Investigation, Writing – original draft, Project administration. **Wilson Cheong Hin Hong:** Writing – review & editing. **Erdan Ma:** Conceptualization, Validation, Writing – review & editing. **Cindia Ching Chi Lam:** Conceptualization, Methodology, Software, Data curation, Formal analysis. **Leyi Zhao:** .

## Declaration of Competing Interest

The authors declare that they have no known competing financial interests or personal relationships that could have appeared to influence the work reported in this paper.

## Data Availability

Data on how job satisfaction and perceived value of CSR demotivate undesirable job habits during crisis (Original data) (Mendeley Data)
